# Supplementary material for: COVID-19 Is an Independent Risk Factor for Detrimental Invasive Fungal Disease in Patients on Veno-Venous Extracorporeal Membrane Oxygenation: A Retrospective Study
Source: J Fungi (Basel). 2023 Jul 15;9(7):751. doi: 10.3390/jof9070751 (PMC10381551; doi:10.3390/jof9070751)
Supplement: Supplementary file 1 [file jof-09-00751-s001.zip › jof-2449328-supplementary.pdf]

Suppl. Table 1. Pre-existing Comorbidities Indexed in the Charlson Comorbidity Index (CCI). AIDS: Acquired Immuno-Deficiency Syndrome. *Pearson's Chi-squared test. §Fisher's Exact test for count data.

COVID-19 Status (n)	Total (452)	"non-COVID-19" (334)	"COVID-19" (118)	p
Myocardial infarction (yes (%))	36 (8)	30 (9)	6 (5)	0.179*
Congestive Heart Failure (yes (%))	36 (8)	32 (10)	4 (3)	0.033*
Peripheral Vascular Disease (yes (%))	31 (7)	30 (9)	1 (1)	0.003*
Cerebrovascular Disease (yes (%))	46 (10)	44 (13)	2 (2)	<0.001*
Dementia (yes (%))	3 (1)	2 (1)	1 (1)	1.000§
Chronic Pulmonary Disease (yes (%))	127 (28)	108 (32)	19 (16)	<0.001*
Connective Tissue Disease (yes (%))	9 (2)	8 (2)	1 (1)	0.457§
Peptic Ulcer Disease (yes (%))	11 (2)	11 (3)	0 (0)	0.074§
Mild Liver Disease (yes (%))	24 (5)	21 (6)	3 (3)	0.119*
Severe Liver Disease (yes (%))	6 (1)	6 (2)	0 (0)	0.347§
Diabetes without Organ Damage (yes (%))	72 (16)	48 (14)	24 (20)	0.128*
Diabetes with Organ Damage (yes (%))	28 (6)	25 (7)	3 (3)	0.056*
Hemiplegia (yes (%))	4 (1)	4 (1)	0 (0)	0.577§
Severe Kidney Disease (yes (%))	38 (8)	32 (10)	6 (5)	0.130*
Any Tumor (yes (%))	36 (8)	32 (10)	4 (3)	0.033*
Leukemia (yes (%))	8 (2)	7 (2)	1 (1)	0.687§
Lymphoma (yes (%))	10 (2)	9 (3)	1 (1)	0.466§
Metastatic Tumor (yes (%))	7 (2)	7 (2)	0 (0)	0.198§
AIDS (yes (%))	2 (0.4)	2 (1)	0 (0)	1.000§

Suppl. Table 2. COVID-19 Cases and IFD According to Pandemic Phase.

Phase	Designation	Start (month/year)	End (month/year)	Cases (total)	Cases (with IFD)	Proportion (with IFD/total)
0	Sporadic cases	01/2020	02/2020	0	0	0%
1	First wave	03/2020	05/2020	6	2	33.3%
2	Summer plateau	05/2020	09/2020	4	0	0%
3	Second wave	09/2020	02/2021	37	6	16.2%
4	Third wave	03/2021	06/2021	30	3	10%
5	Summer plateau	06/2021	08/2021	3	0	0%
6	Fourth wave	08/2021	12/2021	30	2	6.7%
7	Fifth wave	12/2021	Not defined	8	0	0%
